# Calpain activation and disturbance of autophagy are induced in cortical neurons *in vitro* by exposure to HA/*β*-Ga_2_O_3_:Cr^3+^ nanoparticles

**DOI:** 10.7717/peerj.4365

**Published:** 2018-02-07

**Authors:** Yu Lei, Chengkun Wang, Quan Jiang, Xiaoyi Sun, Yongzhong Du, Yaofeng Zhu, Yingmei Lu

**Affiliations:** 1 College of Pharmaceutical Sciences, Zhejiang Unviersity, Hangzhou, Zhejiang Province, China; 2 School of Medicine, Zhejiang University City College, Hangzhou, Zhejiang Province, China; 3 Key Laboratory of Advanced Textile Materials and Manufacturing Technology of the Ministry of Education, Zhejiang Sci-Tech University, Hangzhou, Zhejiang Province, China

**Keywords:** Calpain, Autophagy, Neurotoxicity, Inflammation, Nanoparticles, HA/β-Ga_2_O_3_:Cr^3+^

## Abstract

The toxicity of engineered nanoparticles remains a concern. The knowledge of biohazards associated with particular nanoparticles is crucial to make this cutting-edge technology more beneficial and safe. Here, we evaluated the toxicity of Ga_2_O_3_ nanoparticles (NPs), which are frequently used to enhance the performance of metal catalysts in a variety of catalytic reactions. The potential inflammatory signaling associated with the toxicity of HA/β-Ga_2_O_3_:Cr^3+^ NPs in primary cortical neurons was examined. We observed a dose-dependent decrease in cell viability and an increase in apoptosis in neurons following various concentrations (0, 1, 5, 25, 50, 100 µg/ml) of HA/β-Ga_2_O_3_:Cr^3+^ NPs treatment. Consistently, constitutively active forms of calcineurin (48 kDa) were significantly elevated in cultured primary cortical neurons, which was consistent with calpain activation indicated by the breakdown products of spectrin. Moreover, HA/β-Ga_2_O_3_:Cr^3+^ NPs result in the elevation of LC3-II formation, SQSTM/p62, and Cathepsin B, whereas phosphorylation of CaMKII (Thr286) and Synapsin I (Ser603) were downregulated in the same context. Taken together, these results demonstrate for the first time that calpain activation and a disturbance of autophagy signaling are evoked by exposure to HA/β-Ga_2_O_3_:Cr^3+^ NPs, which may contribute to neuronal injury *in vitro*.

## Introduction

Nanotechnologies have been steadily growing and are used for the delivery of therapeutic material, leading to new important applications in health and life-science (Andrews & Weiss, 2012; Lu et al., 2014a; Lu et al., 2014b; Huang et al., 2013). Nanoscience allows for new approaches in medical intervention, and it may revolutionize established clinical practices (Wagner et al., 2006). For instance, nanoparticles (NPs) represent a promising drug delivery system for treating brain disease (Cardoso et al., 2010; Wang et al., 2016a; Wang et al., 2016b; Ying et al., 2014). However, safety issues related to NPs exposure continue to be debated.

The use of engineered NPs in neurology involves innovative pharmacological strategies, and the blood-brain barrier (BBB) is a defense mechanism against potentially neurotoxic molecules and structures (Barbu et al., 2009). The application of NPs to the central nervous system (CNS) is at an early stage, and the toxic effects of NPs observed in *in vitro* models of neural cells have been scarce (Landsiedel et al., 2012). Additionally, Fe_4_O_3_ NPs showed promise for *in vitro* applications but were subsequently discarded after exhibiting toxicity when utilized *in vivo.* Therefore, these NPs are now limited to *in vitro* diagnostics (Win-Shwe & Fujimaki, 2011; Zhang et al., 2016; Bao et al., 2014; Haute & Berlin, 2017). Another type of NPs, gold ultrafine particles (UFPs; <100 nm), exhibited neurotoxicity by causing transient microglia activation and the induction of Toll-like receptor 2 (TLR-2) promoter activity in transgenic mice (Elder et al., 2006; Hutter et al., 2010). The neurotoxicity of NPs generally includes an inflammation response, DNA damage, cell death (Shin et al., 2010; Yang & Klionsky, 2010). Examples of neuronal damage observed in *in vivo* experiments include damage to the nuclear factor kappa-light-chain-enhancer of activated B cells (NF-κB) and inducible nitric oxide synthases (iNOS) in the cortex exposed to air with high concentrated metal NPs. BBB injury has also been observed (Calderon-Garciduenas et al., 2008; Castranova, 2004; Tian & Lawrence, 1996). For these reasons, the safety of NPs intended for biomedical applications should be carefully evaluated for each new type of engineered NPs (Rivet et al., 2012).

Gallium oxide (Ga_2_O_3_) is a wide band gap semiconductor that is applied in optical components and optical catalysis, for example, catalytic reactions involving CO, H_2_ and methanol (Reddy, Ko & Yu, 2015). Ga_2_O_3_ has multiple crystal forms, including α, β, γ, ε, δ. The β crystal form is the most stable among them, and adding a chromium ion (Cr^3+^) when preparing Ga_2_O_3_ (β-Ga_2_O_3_: Cr^3+^) NPs enables the NPs to emit fluorescence and near-infrared persistent luminescence (Wang et al., 2015a; Wang et al., 2015b). β-Ga_2_O_3_:Cr^3+^ NPs modified with hyaluronic acid (HA) could effectively absorb antineoplastic drugs, and they were shown to bind with CD44, which is highly expressed in tumor cells and is primarily distributed at the tumor site (Ganguly et al., 2016). HA/β-Ga_2_O_3_:Cr^3+^ NPs have been used as drug carriers to deliver anti-cancer drugs, and it was found that fabricated HA/β-Ga_2_O_3_: Cr^3+^/DOX could be taken up by HeLa and MCF-7 cells (Wang et al., 2015a; Wang et al., 2015b). In this study, the cell viabilities of both MCF-7 and Hela were all above 80% even when the concentration of HA/β-Ga_2_O_3_:Cr^3+^ NPs was up to 1,000 µg/ml. And the final concentration of NPs used as a drug carrier is 40 µg/ml. Nevertheless, for the successful translation from bench to bedside, a promising drug delivery system should have low neuronal toxicity.

We here determined the impact of HA/β-Ga_2_O_3_:Cr^3+^ NPs on neurons. Using the SH-SY5Y cell line and primary cortical neurons in combination with pharmacological methods, in an effort to investigate the cell injury-related signaling following the HA/β-Ga_2_O_3_:Cr^3+^ NPs exposure. Our results provide evidence to implicate the role of calpain and autophagy in HA/β-Ga_2_O_3_:Cr^3+^ NPs-induced neurotoxicity.

## Materials and Methods

### Reagents

All chemicals were purchased from Sigma-Aldrich Chemical Co. (St. Louis, MO, USA) unless otherwise noted. Sodium hyaluronate (HA, 95%) and Gallium Oxide (Ga_2_O_3_, 99.99%), were purchased from Aladdin (Shanghai, China).

### Preparation of Ga_2_O_3_ NPs

The preparation and characterization of HA/β-Ga_2_O_3_:Cr^3+^ NPs were carried out as described as previously reported (Wang et al., 2015a; Wang et al., 2015b). The particle size of HA/β-Ga_2_O_3_:Cr^3+^ NPs in the dispersion was determined with a Zetasizer (3000HS; Malvern Instruments, UK). The zeta potential was determined using a Zetaplus/90plusZeta potential and laser particle size analyzer (Brookhaven Instruments, Holtsville, NY, USA) after 100-fold dilutions of the prepared dispersion were made with distilled water (Wang et al., 2015a; Wang et al., 2015b). Samples from three independent experiments performed in triplicate.

### Cell culture and HA/*β*-Ga_2_O_3_:Cr^3+^ NPs treatment

SH-SY5Y cells (human neuroblastoma) were purchased from American Type Culture Collection (ATCC, CRL2266) and cultured in Dulbecco’s modified Eagle’s medium (DMEM, Gibco, Carlsbad, CA, USA) with 10% fetal bovine serum (FBS, Gibco, Carlsbad, CA, USA). Cells were grown in a 5% CO_2_ incubator at 37 °C, and experiments were carried out when the cells reached 80% confluence.

Primary cortical neurons were obtained from 17-day-old embryonic mice and cultured in neurobasal medium (Gibco, Carlsbad, CA, USA) with 2% B27, 1% antibiotics and 0.25% GlutaMAX™ Supplement (Gibco, Carlsbad, CA, USA) for 10 days (Takano et al., 2017). The cells were cultured in a 5% CO_2_ incubator at 37 °C. Then, the cells were seeded in 6-well plates at a density of 1 × 10^6^ cells/mL and treated with HA/β-Ga_2_O_3_:Cr^3+^ NPs at various concentrations from 1 to 100 µg/mL for 12 h before harvesting.

### MTT assay

The cell viability were monitored by a 3-(4,5-dimethylthiazol-2-yl)-2,5-diphenyltetrazolium bromide (MTT) assay with or without HA/β-Ga_2_O_3_:Cr^3+^ NPs exposure (Liu et al., 2010; Wang et al., 2015a; Wang et al., 2015b). Cells were seeded in 96-well plates and cultured at 37 °C in DMEM medium with 10% FBS. When reached 80% confluence, cells were treated with HA/β-Ga_2_O_3_:Cr^3+^ NPs for 12 h. The medium was replaced with 100 µL of MTT (5 mg/mL), and incubated at 37 °C for 4 h. Then, added 200 µL DMSO to each well and incubated at room temperature for 15 min. The absorbance was detected with an automatic microplate reader (DTX880; Beckman Coulter, Brea, CA, USA).

### Western blotting

Harvested primary cortical neurons were used to detect the expression level of proteins related to neuron synapse structures and functions. Western blotting was carried out by SDS polyacrylamide gel electrophoresis (SDS-PAGE) as described previously (Wang et al., 2015a; Wang et al., 2015b; Wang et al., 2017a). The proteins were probed with the primary antibodies: spectrin (1:2,000; Millipore, Billerica, MA, USA), Calcineurin (1:1,000; Abcam, Cambridge, UK), SQSTM/p62 (1:2,000; Abcam, Cambridge, UK), Cathepsin B (1:1,000; Abcam, Cambridge, UK), LC3 (1:2,000; Sigma, St. Louis, MO, USA), PSD95 (1:3,000; Cell Signaling Technology, Danvers, MA, USA), P-CaMKII (1:2,000; Abcam, Cambridge, UK), P-Synapsin I (1:3,000; Millipore, Billerica, MA, USA), P-GluR1 (1:2,000; Millipore, Billerica, MA, USA), Synapsin I (1:3,000; Millipore, Billerica, MA, USA) and β-actin (1:5,000; Hangzhou Dawen Biotech Co., Ltd., Hangzhou, China).

### Confocal immunofluorescence staining and analysis

Immunofluorescence changes of Cathepsin B were examined by confocal microscopy (Han et al., 2011a). Briefly, cells seeded on coverslips were washed 3 times in PBS and fixed in 4% formaldehyde. The cells were then incubated with antibodies against Cathepsin B (rabbit polyclonal antibody; Abcam, Cambridge, UK) overnight at 4 °C, followed by incubation with secondary antibodies conjugated to Alexa fluor 488 (PerkinElmer Life Sciences, Boston, MA). Immunofluorescence was visualized using a Nikon A1R confocal microscope.

### TUNEL assay

Apoptosis was analyzed using *In Situ* Cell Death Detection Fluorescein (11684795910; Roche, Basel, Switzerland) following the manufacturer’s instructions (Jiang et al., 2017). Images were recorded by using a Nikon A1R confocal microscope after counterstaining with DAPI.

### ROS detection

A fluorescent probe, 2′, 7′-dichlorofluorescein diacetate (Sigma, D6883), was used to examine the ROS levels in SH-SY5Y cells after exposure to NPs. Cells were cultured on glass coverslips overnight and then treated with various doses of NPs for 12 h. Before being fixed in 4% formaldehyde, cells were incubated with 2′, 7′-dichlorofluorescein diacetate for one hour (Wang et al., 2017b). Nucleus were stained with DAPI after fixation, and the probe fluorescence in SH-SY5Y cells was visualized by confocal microscopy (Nikon A1R).

### Data and statistical analysis

The significance of the differences between more than two groups was determined using a one-way ANOVA. The significance of the differences between two groups was determined using a *t*-test. These differences were considered to be significant at *P* < 0.05. All data are expressed as the mean ± S.E.M.

## Results

### HA/*β*-Ga_2_O_3_:Cr^3+^ NPs-induced changes in cell morphology and reduced cell viability

**Figure 1:**
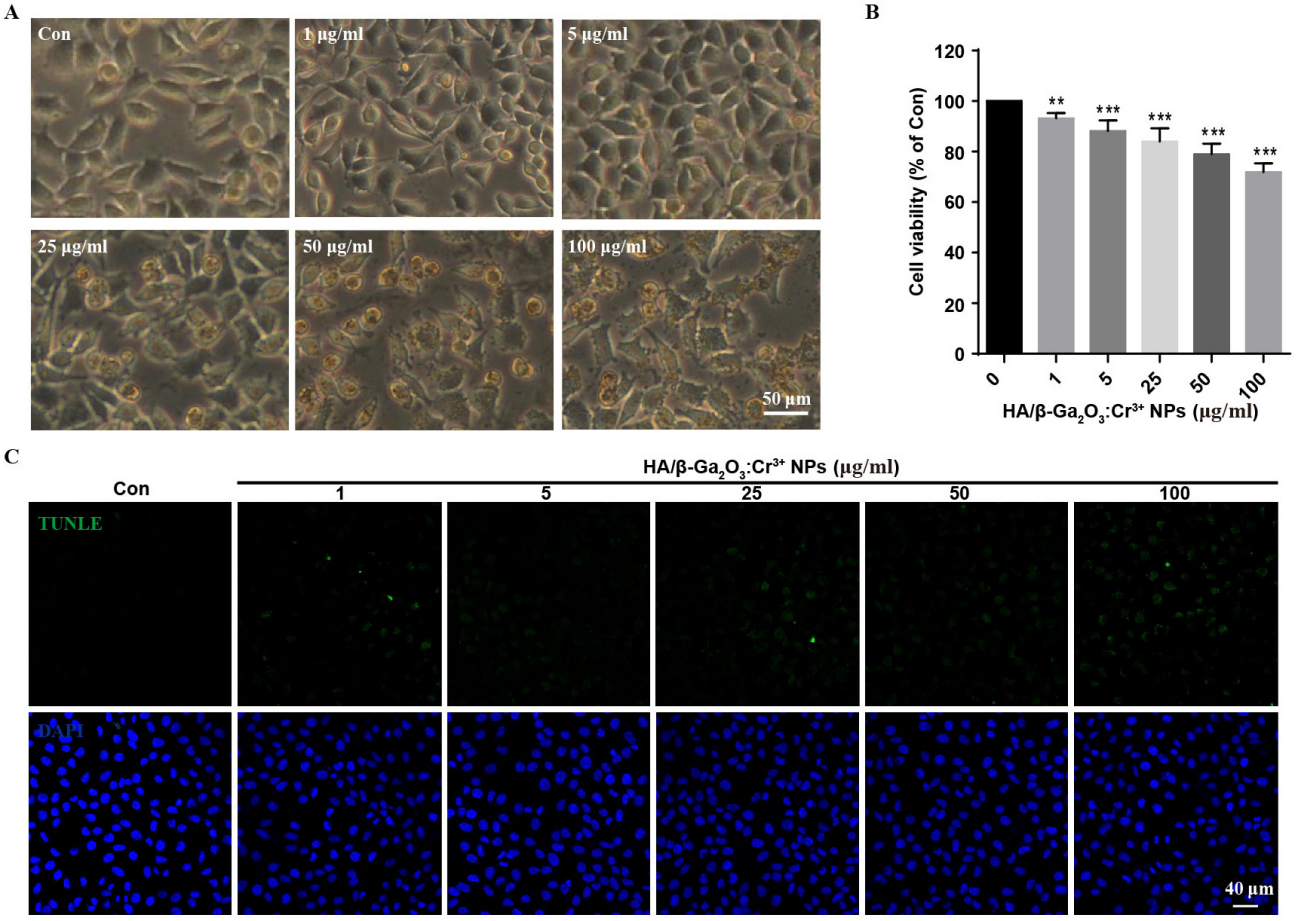
The changes of cell morphology and viability after exposure to HA/*β*-Ga_2_O_3_:Cr^3+^ NPs in SH-SY5Y cell line. (A) The changes of cell morphology after exposure to various doses of HA/*β*-Ga_2_O_3_:Cr^3+^ NPs for 12 h, scale bar = 50 µm. (B) MTT results of SH-SY5Y cells treated with HA/*β*-Ga_2_O_3_:Cr^3+^ NPs, **P < 0.01, ***P < 0.001 versus control group. (C) The results of TUNEL in SH-SY5Y cells after incubation with HA/*β*-Ga_2_O_3_:Cr^3+^ NPs, scale bar = 40 µm.

First, the stability of HA/β-Ga_2_O_3_:Cr^3+^ NPs in different kinds of culture media was detected. As shown in Fig. S1, no significant difference was observed in the size distribution of NPs after incubated in medium with or without FBS for 12 h. SH-SY5Y cells were observed with an inverted fluorescence microscope after incubation with various concentrations (0, 1, 5, 25, 50, 100 µg/mL) of HA/β-Ga_2_O_3_:Cr^3+^ NPs for 12 h. The results showed that cell morphology of neurons was normal at concentrations lower than 5 µg/mL, but cell deformation was observed at concentrations higher than 25 µg/mL (Fig. 1A). The cell viability relative to the control group after exposure to 1, 5, 25, 50, 100 µg/ml HA/β-Ga_2_O_3_:Cr^3+^ NPs for 12 h was 93.18%, 88.15%, 83.94%, 78.95%, 71.86% respectively, indicating that the cell viability was decreased in a concentration-dependent manner after treatment with HA/β-Ga_2_O_3_:Cr^3+^ NPs (Fig. 1B, **P < 0.01, ***P < 0.001). The results of NPs toxicity were further confirmed using the TUNEL assay (Fig. 1C). Consistently, the number of TUNEL^+^ cells relative to DAPI^+^ cells was increased in a dose-dependent manner after exposure to HA/β-Ga_2_O_3_:Cr^3+^ NPs.

### HA/*β*-Ga_2_O_3_:Cr^3+^ NPs decreased the phosphorylation of CaMKII in neurons

**Figure 2:**
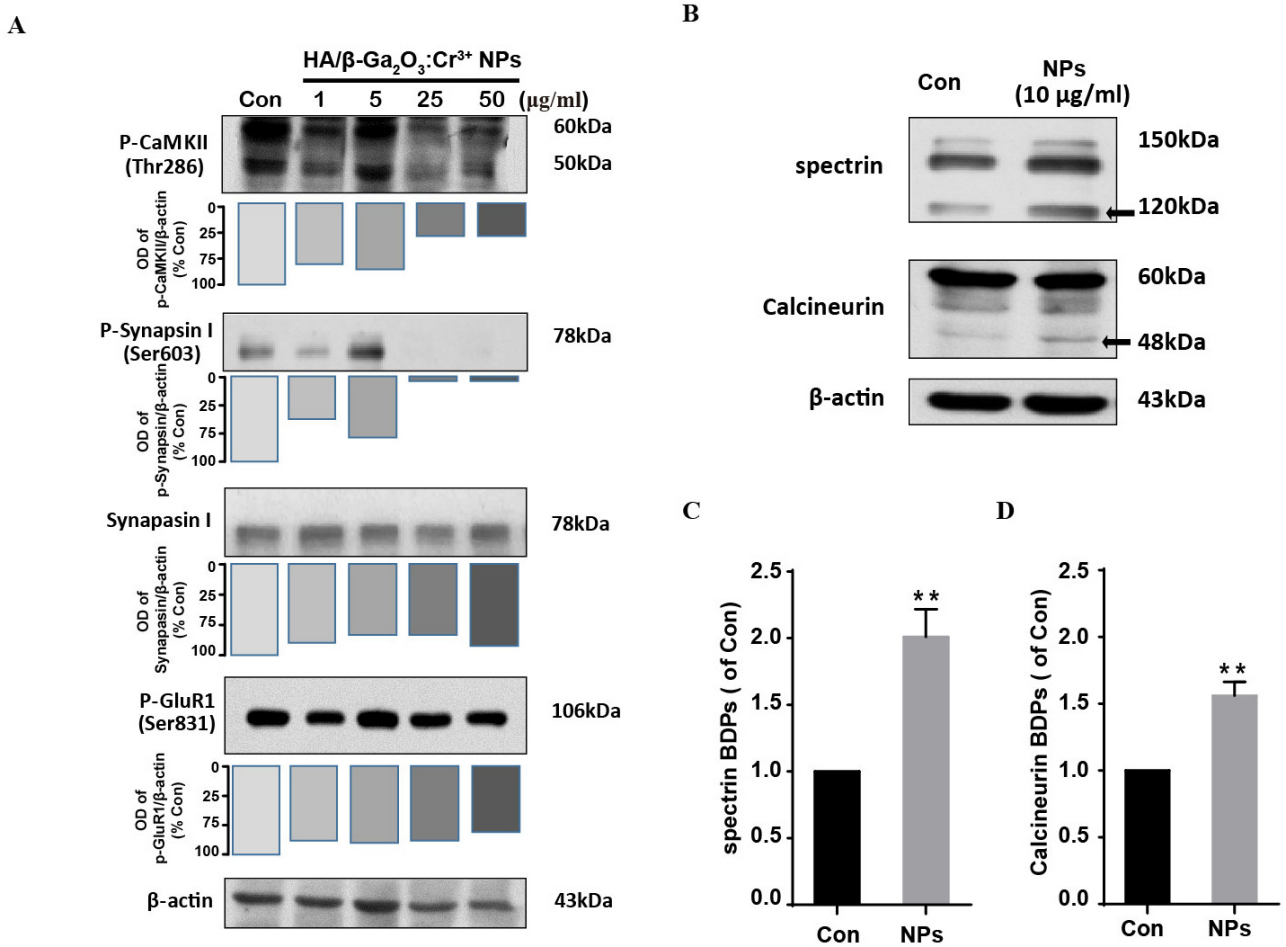
The expression level of proteins associated with synaptic structures and functions in mouse primary cortical neurons. The primary cortical neurons were cultured for 10 days and treated with HA/*β*-Ga_2_O_3_:Cr^3+^ NPs for 12 h. (A) The representative Western blot images of P-CaMKII, P-Synapsin I, Synapsin I and P-GluR 1 in primary cortical neurons after incubation with various doses of HA/*β*-Ga_2_O_3_:Cr^3+^ NPs. (B) The representative data of spectrin and calcineurin in primary cortical neurons after treated with 10 µg/mL HA/*β*-Ga_2_O_3_:Cr^3+^ NPs for 12 h, *β*-actin served as the loading control. Quantification of data on the breakdown products (BDPs) of spectrin (C) and calcineurin (D) from (B). The data are expressed as the mean ± SEM for three independent experiments. **P < 0.01 versus control group.

We next investigate the neuronal toxicity of HA/β-Ga_2_O_3_:Cr^3+^ NPs using primary cortical neurons from wild-type mice. CaMKII is enriched at synapses where it plays a critical role in regulating synaptic transmission (Li et al., 2017; Liu et al., 2007). Here, the phosphorylation of GluR 1 (Ser831), CaMKII (Thr286) and Synapsin I (Ser603) were assessed by Western blotting in primary cortical neurons following HA/β-Ga_2_O_3_:Cr^3+^ NPs treatment for 12 h. Phospho-CaMKII (Thr286) was significantly decreased after treated with 25 and 50 µg/mL HA/β-Ga_2_O_3_:Cr^3+^ NPs (Fig. 2A). Consistently, the expression level of phospho-Synapsin I (Ser603) was markedly decreased in the 25 and 50 µg/mL NPs treatment groups. There was no significant change in the phosphorylation of the postsynaptic AMPA receptor subtype GluR 1 after exposure to various concentrations of NPs. These data suggested that exposure to HA/β-Ga_2_O_3_:Cr^3+^ NPs may interfere with the functions of cortical neurons.

### HA/*β*-Ga_2_O_3_:Cr^3+^ NPs exposure leads to calpain activation

Ca^2+^ signaling activates the apoptotic pathway through the activation of calpain followed by the cleavage of calpain substrates, such as calcineurin (Mahmood et al., 2017). We investigated whether HA/β-Ga_2_O_3_:Cr^3+^ NPs treatment also led to the activation of calpain. Compared to the control group, the level of calcineurin cleavage fragment (48 kDa) was increased after cells were incubated with HA/β-Ga_2_O_3_:Cr^3+^ NPs (Figs. 2B and 2D, *P* < 0.01). It has been established that calcium-sensitive proteases such as calpain can lead to increased spectrin cleavage. Here, HA/β-Ga_2_O_3_:Cr^3+^ NPs treatment resulted in calpain activation, as shown by the elevated levels of spectrin breakdown products (120 kDa) (Figs. 2B and 2C, *P* < 0.01).

### HA/*β*-Ga_2_O_3_:Cr^3+^ NPs disturbed autophagy signaling

**Figure 3:**
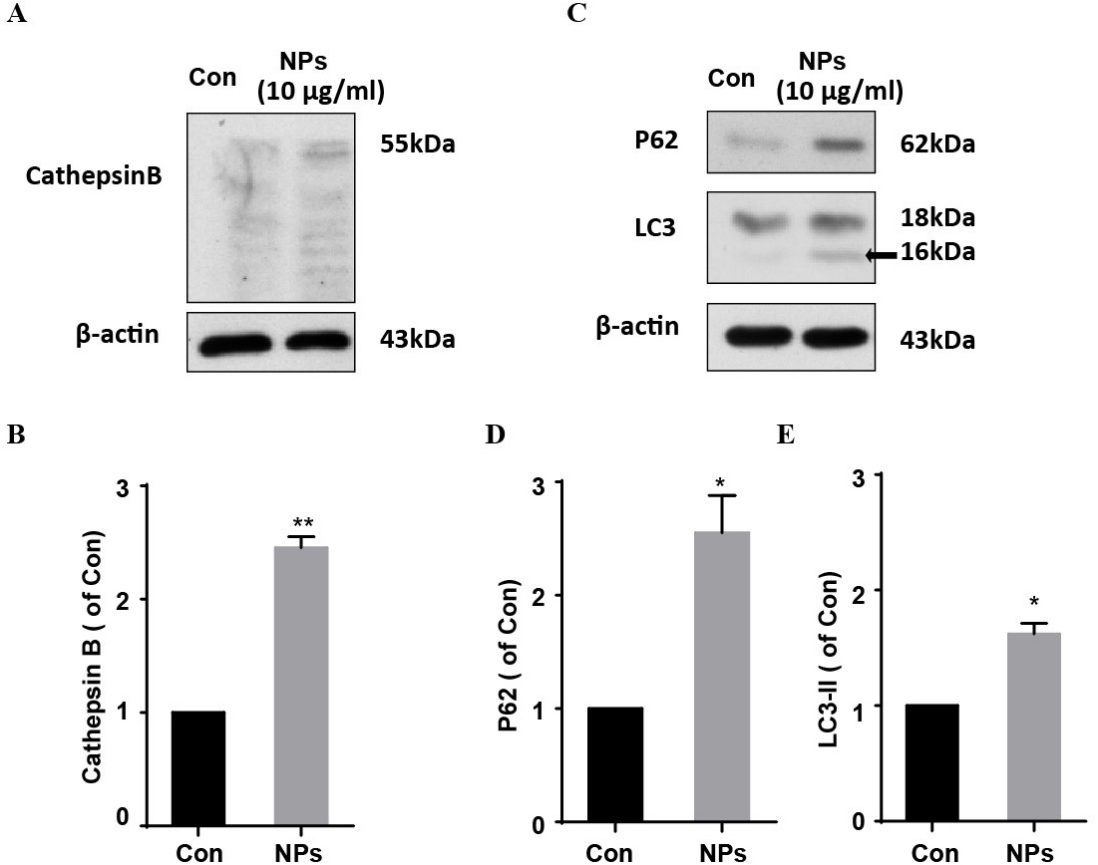
The effects of HA/*β*-Ga_2_O_3_:Cr^3+^ NPs on the autophagy signaling pathway. (A) The cultured primary cortical neurons were incubated with 10 µg/mL HA/*β*-Ga_2_O_3_:Cr^3+^ NPs for 12 h, and intracellular Cathepsin B was measured by Western blot analysis. Immunodetection of *β*-actin was used as a loading control. (B) Quantification of data on Cathepsin B from (A). The data are expressed as the mean ± SEM for three independent experiments, **P < 0.01 versus the control group. (C) The representative Western blot images of LC3 and P62 in primary cortical neurons after incubation with 10 µg/mL HA/*β*-Ga_2_O_3_:Cr^3+^ NPs. Quantification of data on P62 (D) and type II LC3 (16 kDa) (E) from (C), β-actin served as the loading control. The data are expressed as the mean ± SEM for three independent experiments. *P < 0.05, **P < 0.01 versus the control group.

**Figure 4:**
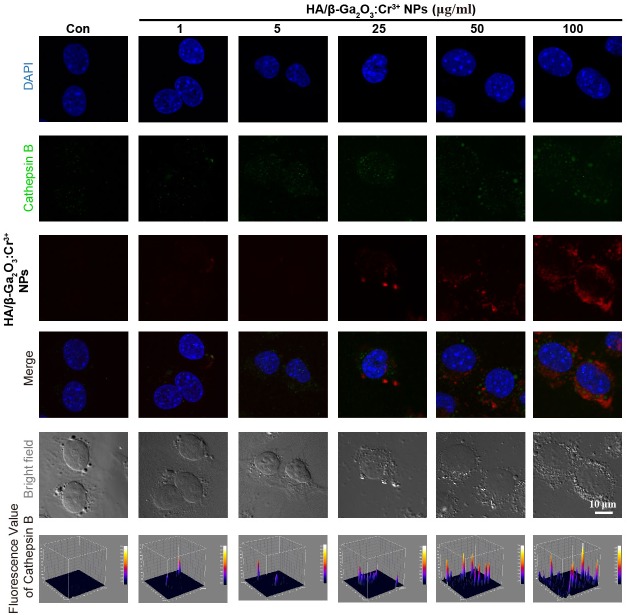
Immunocytochemical analysis shown the uptaken of HA/β-Ga_2_O_3_:Cr^3+^ NPs and the activation of Cathepsin B in SH-SY5Y cells. The excitation wavelength of DAPI, Cathepsin B, and HA/β-Ga_2_O_3_:Cr^3+^ NPs is 405 nm, 488 nm, and 641 nm, respectively. Scale bar = 10 µm.

Dysfunction of autophagic pathways plays a major role in the pathogenic process of cell injury (Han et al., 2011a; Jiang et al., 2017). We found that the expression of mature Cathepsin B was increased in primary cortical neurons after treatment with HA/β-Ga_2_O_3_:Cr^3+^ NPs, compared to the control group (Figs. 3A and 3B, *P* < 0.01). As shown in Figs. 3C–3E, the expression of type II LC3 (16 kDa) was significantly elevated, as well as the level of p62 (*P* < 0.05). Taken together, these results indicated that the fusion of autophagosomes and lysosomes in the autophagy flux may be blocked in primary cortical neurons after treatment with HA/β-Ga_2_O_3_:Cr^3+^ NPs for 12 h, thereby resulting in the aberrant accumulation of LC3-II and p62. Consistently, the fluorescence intensity of Cathepsin B (green) was significantly enhanced in a concentration-dependent manner following treatment with HA/ β-Ga_2_O_3_:Cr^3+^ NPs (Fig. 4). We also used 2′, 7′-dichlorofluorescein diacetateto detect ROS levels in the cells after incubation with NPs. As shown in Fig. S2, an increase in ROS was observed at a dose as low as 1 µg/mL HA/β-Ga_2_O_3_:Cr^3+^ NPs.

## Discussion

The increasing applications for nanomaterials have led to a growing concern about the bioavailability and toxicity of nano-sized materials (Igarashi, 2008). Herein, we evaluated the molecular mechanism underlying HA/β-Ga_2_O_3_:Cr^3+^ NPs-induced neurotoxicity. Our experimental results showed that HA/β-Ga_2_O_3_:Cr^3+^ NPs at a certain concentration could decrease cell viability and damage the synaptic functions by inducing calpain activation and excessive autophagy.

β-Ga_2_O_3_ has been widely applied in optoelectronic devices by being doped with foreign impurities, which can radiate various photoluminescence spectra from visible to near-infrared light (Nogales et al., 2007; Liu et al., 2008). β-Ga_2_O_3_ doped with Cr^3+^ has been used not only as a fluorescent probe for bio-imaging but also as a drug carrier for biomedicine (Wang et al., 2015a; Wang et al., 2015b). The viability of both MCF-7 and HeLa cells was greater than 80%, even when the concentration of HA/β-Ga_2_O_3_:Cr^3+^ NPs was as high as 1,000 µg/mL (Wang et al., 2015a; Wang et al., 2015b). Pyramidal neurons in the brain are responsible for brain functions, and they are the most sensitive and delicate cells in bio-organisms, which means that they are among the most vulnerable cells to nanoparticles (Paterson-Brown et al., 1986). To our knowledge, studies addressing whether the HA/β-Ga_2_O_3_:Cr^3+^ NPs nanoparticles could induce neuronal toxic effects have not been reported previously. In this study, the cellular morphology, cell viability and TUNEL were examined in the SH-SY5Y cell line and in primary cortical neurons to assess the neuronal toxicity of HA/β-Ga_2_O_3_:Cr^3+^ NPs. The data showed that cell viability relative to the control group after exposure to 1, 5 or 25 µg/mL HA/β-Ga_2_O_3_:Cr^3+^ NPs for 12 h was 93.18%, 88.15%, 83.94%, respectively, indicating that HA/β-Ga_2_O_3_:Cr^3+^ NPs can induce neuronal toxicity even at a low concentration (1 µg/mL). The final concentration of NPs we used as a drug carrier was 40 µg/mL (Wang et al., 2015a; Wang et al., 2015b). From these results, we conclude that neurons are very sensitive to HA/ β-Ga_2_O_3_:Cr^3+^ NPs, and this makes them a prime target for the neurotoxic effects of exogenous HA/β-Ga_2_O_3_:Cr^3+^ NPs.

CaMKII is enriched at the synapse and plays important roles in controlling synaptic strength and plasticity, which are mediated through substrate binding and the intramolecular phosphorylation of holoenzyme subunits (Li et al., 2017; Liu et al., 2007; Navakkode et al., 2017). Paralleled with the down-regulation of phospho-CaMKII (Thr286), phospho-synapsin I (Ser603) was also significantly decreased in the cultured primary cortical neurons following exposure to higher concentrations of HA/β-Ga_2_O_3_:Cr^3+^ NPs (25 and 50 µg/mL) for 12 h, whereas the expression level of total synapsin I did not change. Interestingly, we also observed that phospho-synapsin I (Ser603) levels were transiently elevated following exposure to HA/β-Ga_2_O_3_:Cr^3+^ NPs at 5 µg/mL, and the same phenomenon was also observed for the phosphorylation of CaMKII. This turnover may be interpreted as a compensatory mechanism of the neurons. The activation of CaMKII is closely related to changes in phosphorylation of its post-synaptic and pre-synaptic substrates, such as synapsin I and GluR1 (Liao et al., 2013). However, there was no significant change observed in the phosphorylation of GluR1 following NPs treatment; this may due to the different sensitivity of presynaptic and postsynaptic structures following exposure to HA/β-Ga_2_O_3_:Cr^3+^ NPs.

Accumulating evidence has shown that intracellular Ca^2+^ increases after cells are exposed to a variety of nanoparticles (Dubes et al., 2017; Huang et al., 2009), and this increase has been associated with the inhibition or activation of various intracellular signaling pathways, leading to a cytotoxic effect. These alterations in Ca^2+^ homeostasis lead to persistent, pathologic activation of calpain, which is indicative of cell cytoskeletal injury (Lu et al., 2016). Our results revealed the pathologic activation of calpain following exposure to HA/β-Ga_2_O_3_:Cr^3+^ NPs, detected by increased levels of cleaved fragments of spectrin and calcineurin. The hyperactivation of calpain is implicated in the breakdown of cytoskeletal molecules, such as spectrin, microtubule subunits, microtubule-associated proteins, and neurofilaments (Lu et al., 2016; Wu et al., 2004; Ding et al., 2016). Taken together, these data confirm the role of the calpain pathway as a regulatory mechanism involved in HA/β-Ga_2_O_3_:Cr^3+^ NPs-induced neuronal toxicity.

The homeostasis of autophagy-lysosome signaling plays a critical role in protecting against neurovascular injury (Lu et al., 2014a; Lu et al., 2014b; Han et al., 2011a; Jiang et al., 2017). Based on our results, we could conclude that the disturbance of the autophagy process was also involved in HA/β-Ga_2_O_3_:Cr^3+^ NPs-induced neurotoxicity. It has been reported that NPs-induced autophagy may be an adaptive cellular response, aiding in the degradation and clearance of the nanomaterial; however, overstimulation of autophagy can induce harmful cellular dysfunction (Johnson-Lyles et al., 2010; Li et al., 2010; Neun & Stern, 2011). During the autophagy process, cathepsin B is released into the cytoplasm from lysosomes and is subsequently activated. Therefore it is involved in the activation of inflammatory processes, which result in an increased inflammation response (Han et al., 2011b; Nakashima et al., 2003). Here, the expression of cathepsin B, LC3 II and SQSTM/p62 was significantly increased in primary cortical neurons following incubation with 10 µg/mL HA/β-Ga_2_O_3_:Cr^3+^ NPs for 12 h. The immunocytochemistry results also confirmed the elevation of cathepsin B after exposure to HA/β-Ga_2_O_3_:Cr^3+^ NPs, which was in agreement with the increased uptake of NPs. Since several mechanisms can induce the dysfunction of autophagy flux (Nakashima et al., 2003), we also examined the ROS levels in cells following HA/β-Ga_2_O_3_:Cr^3+^ NPs treatment. Our fluorescence images showed that HA/β-Ga_2_O_3_:Cr^3+^ NPs exposure increased the generation of ROS. Indeed, the accumulation of ROS induced by exposure to NPs can disturb the autophagy flux, ultimately leading to cell death, and it can change neuronal function (Wang et al., 2016a; Wang et al., 2016b). Indeed, biological effects of metal ions (Ga^3+^ or Cr^3+^) are poorly understood and their potential toxicity vary depending on the exposure conditions (Catelas et al., 2003; Huk et al., 2004; Wang et al., 2015a; Wang et al., 2015b; Pourshahrestani et al., 2017). Besides the data for stability of HA/β-Ga_2_O_3_:Cr^3+^ NPs, an important limitation of the present study was that lack of extensive characterization of the solubility and release of the Ga^3+^ or Cr^3+^ over the time course of the experiment. Therefore, a good safety margin should be further studied in an animal model to investigate the neuronal toxicity of HA/β-Ga_2_O_3_:Cr^3+^ NPs in the context of an intact brain with or without risk factors.

## Conclusions

In summary, our results suggested that the exposure of neurons to HA/β-Ga_2_O_3_:Cr^3+^ NPs could induce calpain activation and the disturbance of autophagy signaling, which may result in neuronal damage.

##  Supplemental Information

10.7717/peerj.4365/supp-1 Figure S1 The size distribution of the HA/*β*-Ga_2_O_3_:Cr^3+^ NPs was assessed in different culture media There was no significant difference in the size distribution of NPs in the media with or without 10% FBS.

10.7717/peerj.4365/supp-2 Figure S2 The results of a fluorescent probe, 2′, 7′-dichlorofluorescein diacetate, which was used to detect the ROS level The increased fluorescence means that the ROS level was significantly increased as the doses of HA/β-Ga_2_O_3_:Cr^3+^ NPs was increased, scale bar = 20 µm.

10.7717/peerj.4365/supp-3 Data S1 Raw data

## Additional Information and Declarations

### Data Availability

The following information was supplied regarding data availability: The raw data is provided as a Supplemental File.
