# Supplementary figures and images for: Calpain activation and disturbance of autophagy are induced in cortical neurons in vitro by exposure to HA/β-Ga2O3:Cr3+ nanoparticles
Source: PeerJ. 2018 Feb 7;6:e4365. doi: 10.7717/peerj.4365 (PMC5807884; doi:10.7717/peerj.4365)

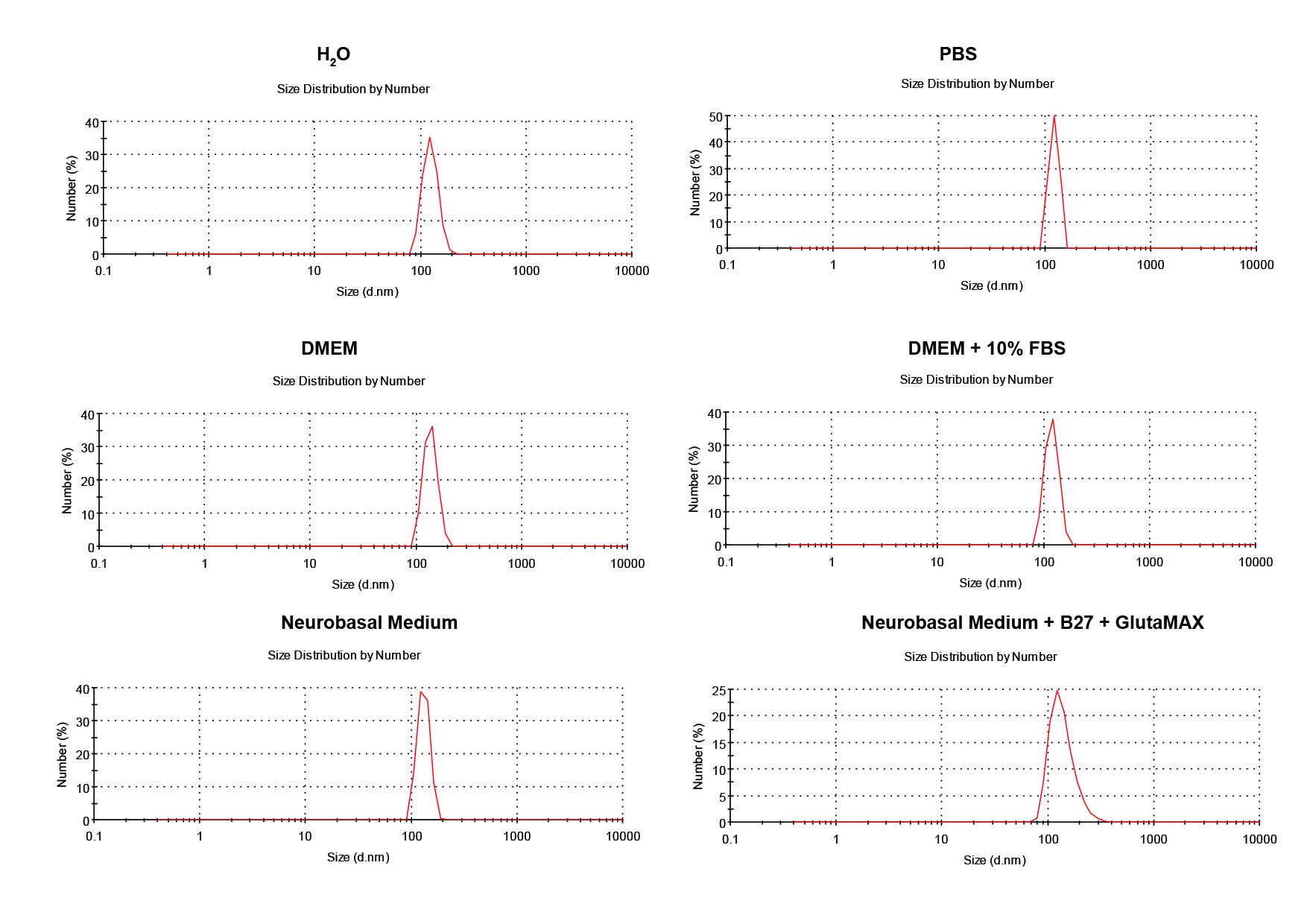

Supplement: Figure S1 — There was no significant difference in the size distribution of NPs in the media with or without 10% FBS. [file peerj-06-4365-s001.jpg]

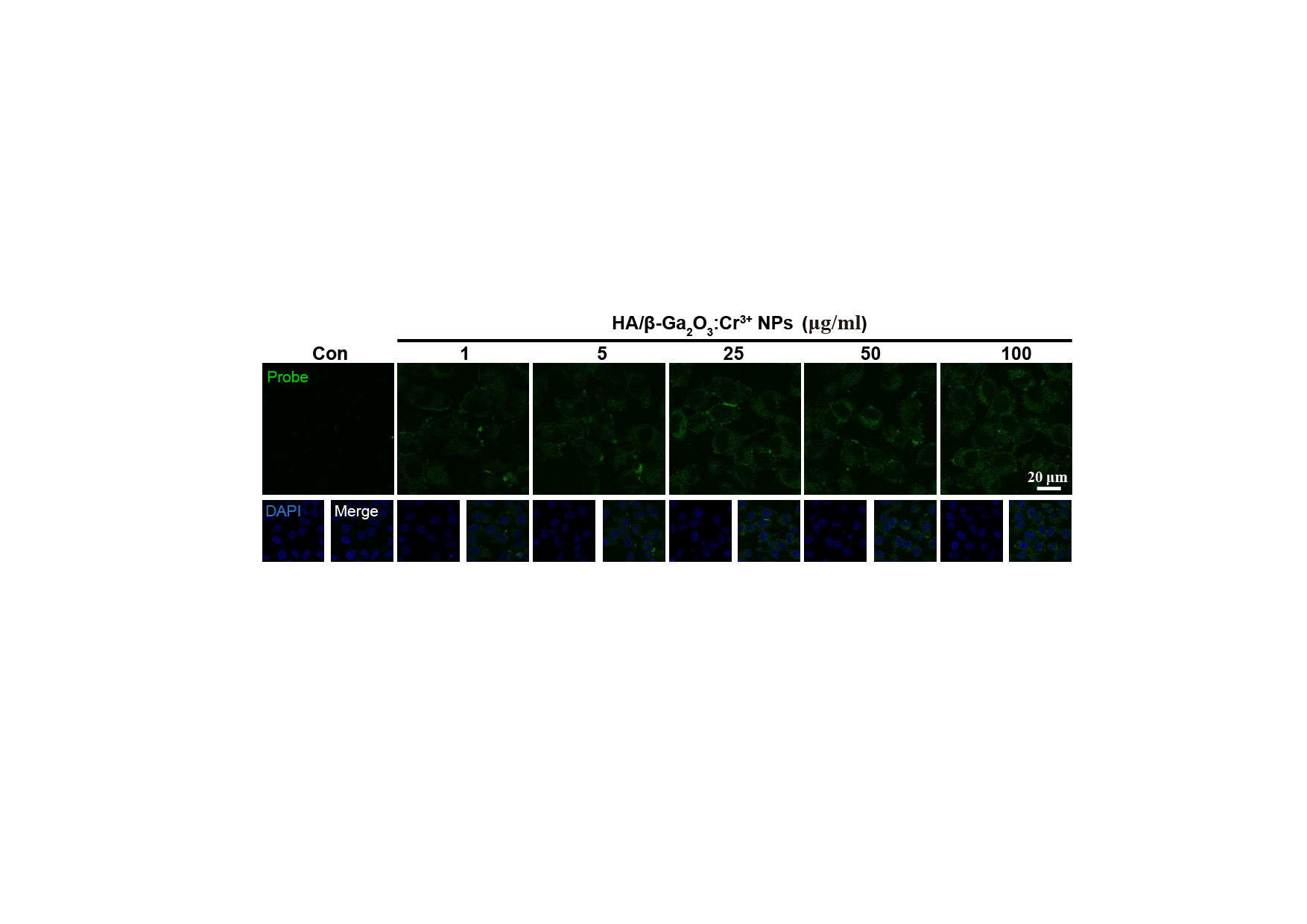

Supplement: Figure S2 — The increased fluorescence means that the ROS level was significantly increased as the doses of HA/β-Ga2O3:Cr3+ NPs was increased, scale bar = 20 µm. [file peerj-06-4365-s002.jpg]

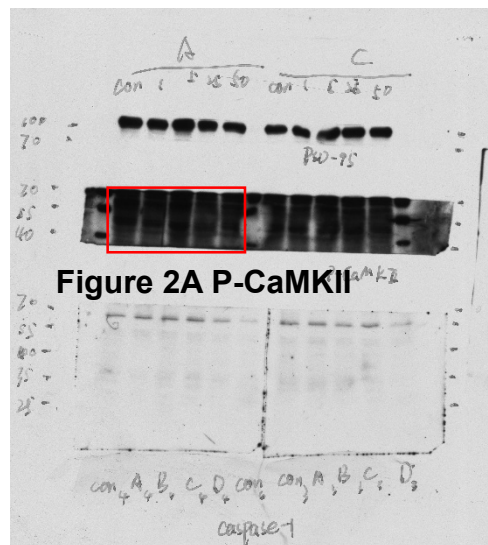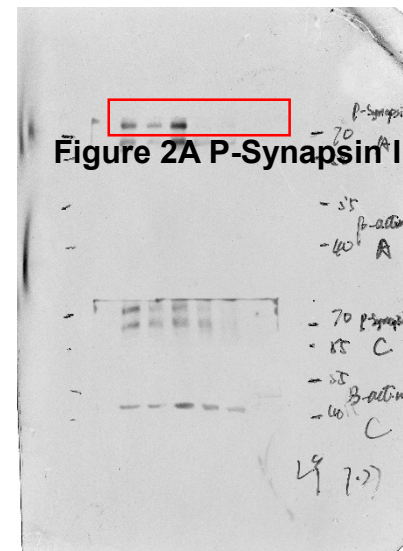

Figure 2B spectrin

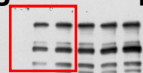

Figure 2B Calcineurin

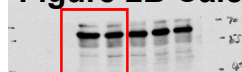

Figure 2B  $\beta$ -actin

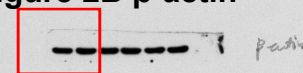

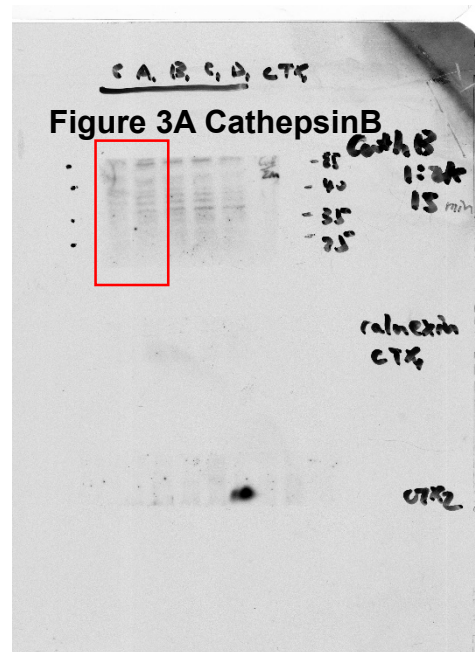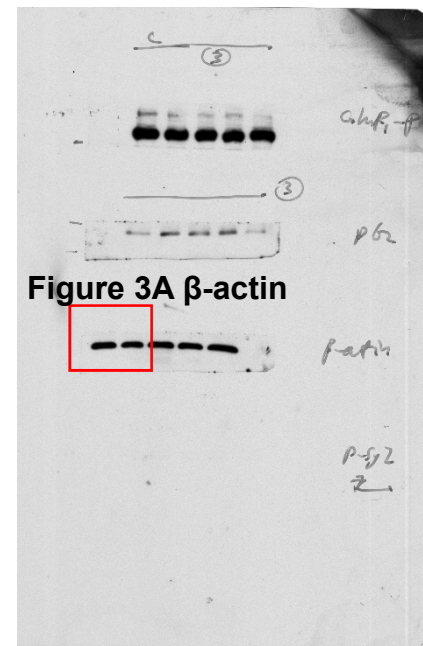

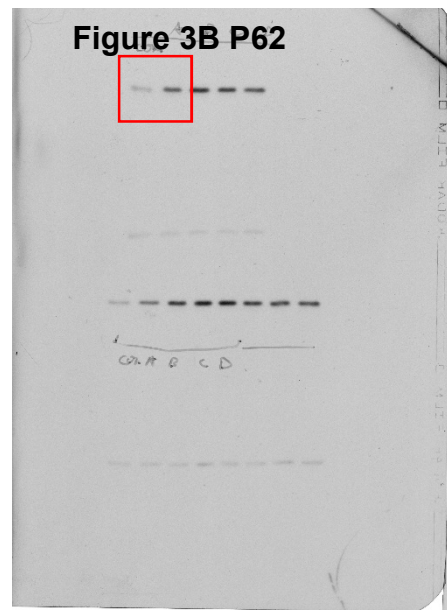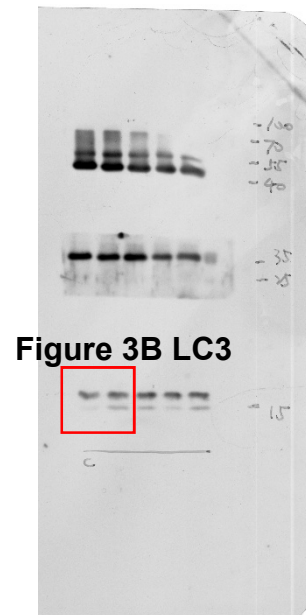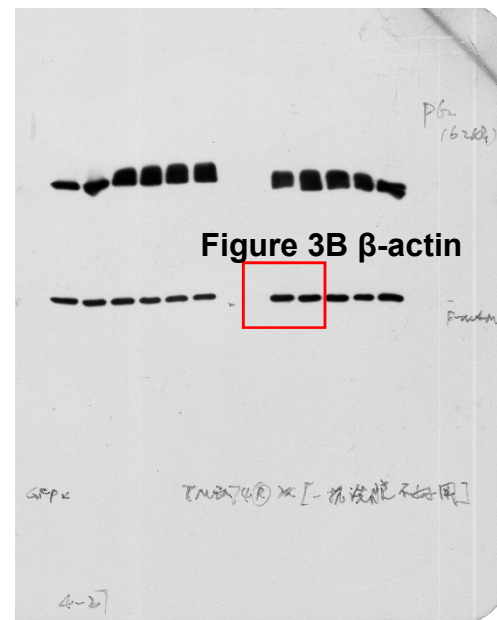

Supplement: Data S1 [file peerj-06-4365-s003.pdf]
